# Identification of sorbitol esterification of glutamic acid by LC-MS/MS in a monoclonal antibody stability assessment

**DOI:** 10.1371/journal.pone.0295735

**Published:** 2024-05-02

**Authors:** Bin Yu, Shannon Williams, Zhengdong Yang, Glen Young

**Affiliations:** Analytical Development Department, Coherus BioSciences, Camarillo, California, United States of America; University of Ghana, GHANA

## Abstract

The stability of monoclonal antibodies (mAbs) is vital for their therapeutic success. Sorbitol, a common mAb stabilizer used to prevent aggregation, was evaluated for any potential adverse effects on the chemical stability of mAb X. An LC-MS/MS based analysis focusing on the post-translational modifications (PTMs) of mAb X was conducted on samples that had undergone accelerated aging at 40°C. Along with PTMs that are known to affect mAbs’ structure function and stability (such as deamidation and oxidation), a novel mAb PTM was discovered, the esterification of glutamic acid by sorbitol. Incubation of mAb X with a 1:1 ratio of unlabeled sorbitol and isotopically labeled sorbitol (^13^C6) further corroborated that the modification was the consequence of the esterification of glutamic acid by sorbitol. Levels of esterification varied across glutamic acid residues and correlated with incubation time and sorbitol concentration. After 4 weeks of accelerated stability with isotopically labeled sorbitol, it was found that 16% of the total mAb possesses an esterified glutamic acid. No esterification was observed at aspartic acid sites despite the free carboxylic acid side chain. This study unveils a unique modification of mAbs, emphasizing its potential significance for formulation and drug development.

## Introduction

The chemical stability of a therapeutic monoclonal antibody (mAb) is dependent on its amino acid composition, conformation, formulation as well as any PTMs. Excipients are added to achieve isotonicity and to stabilize the protein to minimize aggregation and chemical degradation. [1, 2]. Sorbitol is a polyol excipient commonly used in commercial mAb therapeutic products at concentrations ranging from 35–50 mg/mL and thus has been thoroughly evaluated as a formulation component in stability studies [3, 4].

During formulation development, the stability assessment of a mAb under stressed conditions (e.g. 40 °C for several days, weeks and months) is widely used to assist in formulation screening [5]. Despite some limitations in its predictive ability, stressed stability has remained relevant for establishing long term storage conditions [6, 7]. PTMs, including deamidation and oxidation, are often investigated during stability assessments due to the potential impacts on potency, aggregation, and structural conformation [8–12].

In this study, we identified a novel modification involving the esterification of glutamic acid by sorbitol. Sorbitol is a saturated sugar alcohol and generally inert excipient. Although amino acid modification by sorbitol has yet to be reported, free fatty acid esterification by sorbitol to form sorbitol fatty acid esters, which are used as emulsifiers in the cosmetic and food industries [13, 14]. The sorbitol-fatty acid ester formation reaction requires an acidic pH and high temperature (200°C) for several hours or alternatively under mild condition in the presence of a lipase in organic solvents [15, 16]. Therefore, it is possible that low but detectable glutamic acid esterification could occur under stressed conditions (40°C) and long incubation times (4 weeks).

## Material and methods

### Stressed stability studies

The stressed stability study adhered to ICH guideline recommendations (Q5C) for stability testing of biotechnological/biological products [17]. Briefly, 40 mg/mL monoclonal antibody X in 270 mM sorbitol, 10 mM Histidine, 0.01% PS80, pH 5.5 formulation buffer was prepared and incubated at 40 °C for 0 week, 1 week, 2 weeks and 4 weeks. An additional sample with 540 mM sorbitol (1:1 ratio of 270 mM sorbitol and 270 mM isotope labeled sorbitol (^13^C6) from Cambridge Isotope Laboratories (Andover, MA)) in 10 mM Histidine, 0.01% PS80, pH 5.5 formulation buffer was prepared and left at 40 °C for 4 weeks. After preparation, all samples were stored in -80 °C for future analysis.

### Trypsin digestion

After 4 weeks, all previous samples stored in -80 °C were digested using the SMART Digest^™^ Trypsin Kit (Thermo Scientific^™^, San Jose, CA). Briefly, 100 μg of monoclonal antibody was digested with trypsin beads. The filtered solution was mixed with dithiothreitol (Fisher, Hampton, NH) for 30 min to break inter and intra disulfide bonds in the heavy chains (HC) and light chains (LC). Iodoacetamide (Sigma, St. Louis, MO) was added and left in the dark for 30 min to block any free cysteine residues. The reaction was stopped by the addition of trifluoroacetic acid.

### LC-MS/MS methods

An injection of 25 μg of digested sample was made into a Thermo Scientific^™^ Vanquish UHPLC connected to a Thermo Scientific^™^ Orbitrap Fusion Lumos Mass Spectrometer (San Jose, CA). The gradient was run from 0% ACN to 40% over 45 min through a Thermo Scientific^™^ Acclaim^™^ VANQUISH^™^ C18 UHPLC Column (2.1X250mm). Data was collected in data-dependent acquisition mode. Mass spectrometry (MS) resolution was set at 120,000 and tandem mass spectrometry (MS/MS) fragmentation targeted for the top 5 most intense ions at 60,000 resolution. Fragmentation was performed by High-energy Collisional Dissociation (HCD) with energy set at 30%. The ion intensity threshold was set at 1e5 with dynamic exclusion set for 30 seconds when an event occurs 2 times over any 7 seconds period.

### Data analysis software and procedure

Genedata Expressionist^®^ 16.0 (Genedata AG, Basel, Switzerland) was used to analyze the MS data. In addition to commonly screened PTMs (oxidation, deamidation, etc.), the Genedata Expressionist^®^ Wildcard search module was implemented to identify any unassigned modification within a ± 1,000 Da range of their corresponding unmodified species. For any putative esterified peptides, MS and MS/MS spectra were further manually validated by Qual Browser in Thermo Scientific Xcalibur 4.2 (San Jose, CA). The Genedata Expressionist^®^ Extracted Ion Chromatogram based module was used to integrate ion intensity peaks and to calculate modification percentages.

## Results

### Identification of glutamic acid esterification

**Fig 1:**
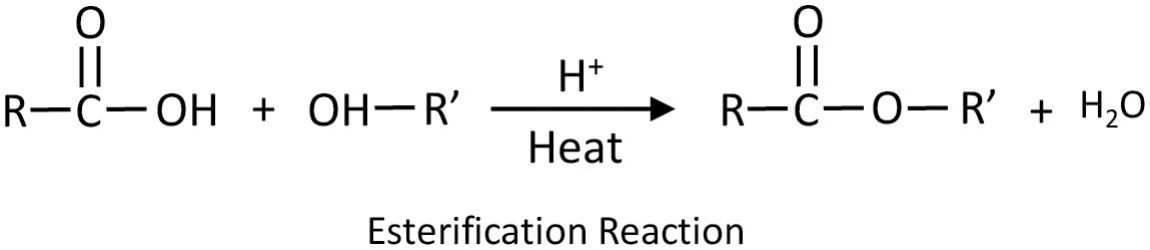
General scheme of the esterification reaction of an acid with a sugar alcohol.

Mass spectrometry data was processed by Genedata Expressionist^®^ to aid in the screening for common and known PTMs. After identifying the more established PTMs with the ‘Peptide Search’ module, an additional search of the data utilizing the ‘Wildcard module’ identified peptides possessing unknown and/or ambiguous modifications. This search screened for any MS/MS spectra that matched a previously identified peptide, but one that also contains an unassigned mass shift. The module reported a +164.07 Da modification at several glutamic acid residues across multiple peptides. This modification was also observed to grow in intensity over longer incubation times. The mass of this potential modification corresponds to the molecular weight of sorbitol after the loss of H_2_O (+164.07 g/mol). Given the carboxylic acid side chain (-COOH) of glutamic acid residues and the alcohol (-OH) groups in sorbitol, we hypothesized that this mass change could be due to esterification of glutamic acid by sorbitol under acidic conditions (Fig 1).

**Fig 2:**
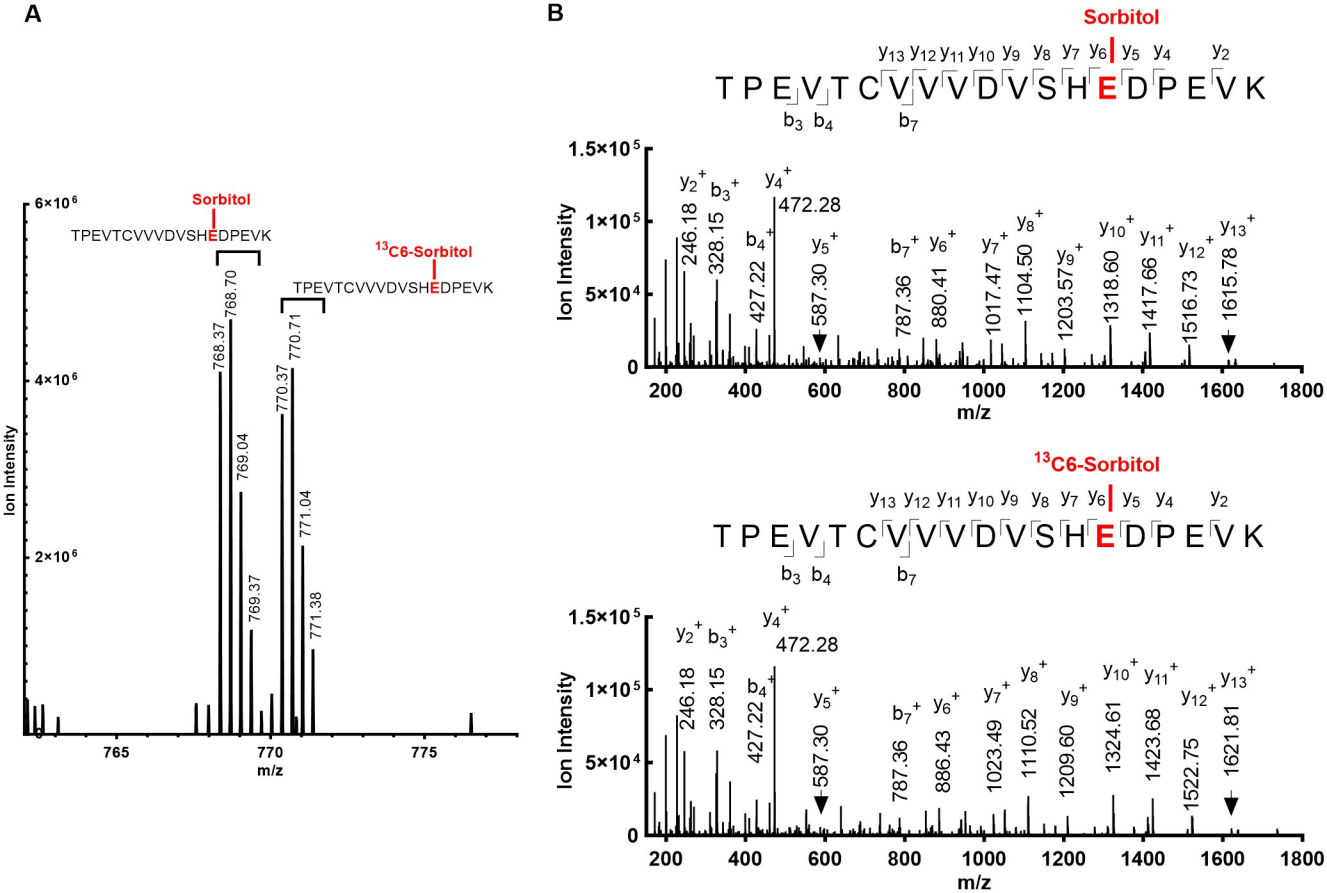
Confirmation of glutamic acid esterification by sorbitol. **(A)** MS spectrum of two species with [M + 3H]^+3^ ions at approximately equivalent intensity. A m/z difference of 2 was observed in the isotopically labeled sorbitol (^13^C6) sample. The difference in the fragments’ molecular weight is from the incorporation of the isotopically labeled sorbitol (^13^C6). **(B)** MS/MS spectra of tryptic peptide HC 263–281 esterified at E276. The two spectra show the TPEVTCVVVDVSHEDPEVK peptide with the same y and b ions (N-terminal and C-terminal fragments of the peptide are marked as y- and b-ions). The lower spectrum however shows a 6 Da mass difference in the y_6_^+^ -y_13_^+^ ions; a consequence of esterification from the isotopically labeled sorbitol (^13^C6) at E276.

To confirm that the +164.07 Da modification is the result of sorbitol esterification, an additional sample was formulated with isotopically labeled sorbitol (^13^C6) mixed 1:1 with unlabeled sorbitol and incubated at 40 °C for 4 weeks. In this sample, we observed the monoisotopic [M + 3H]^+3^ ion of modified TPEVTCVVVDVSHEDPEVK after cysteine alkylation by iodoacetamide at mass-to-charge (m/z) 768.37 (Fig 2A). This modification is consistent with the expected change in mass from esterification by ^12^C6 labeled sorbitol (the theoretical monoisotopic molecular weight of the alkylated peptide prior to esterification is 2302.09 Da). Additionally, an intensity of similar intensity peak at m/z 770.37 was also observed, which is consistent with ^13^C6-sorbitol esterification of the same peptide. These peak pairs at m/z 768.37 and m/z 770.37 reflect the 6 Da difference between ^12^C6 and ^13^C6 sorbitol and substantiate the claim that modification of the TPEVTCVVVDVSHEDPEVK peptide fragment was from sorbitol. Subsequent MS/MS sequencing was performed on each of these ions, confirming that they are both from the TPEVTCVVVDVSHEDPEVK peptide, with identical y_2_^+^, y_4_^+^ and y_5_^+^ mass but multiple y ions from y_6_^+^ to y_13_^+^ reflecting the 6 Da difference of the ^13^C6-sorbitol at E276 (Fig 2B).

### Esterification modification in several tryptic peptides

Multiple esterified glutamic acid residues were identified by LC-MS/MS. In sum, seven tryptic peptides were found to be esterified across both the heavy and light chains. Three of these tryptic peptides contained a single glutamic acid, two tryptic peptides contained two glutamic acids and two tryptic peptides contained three glutamic acids.

**Fig 3:**
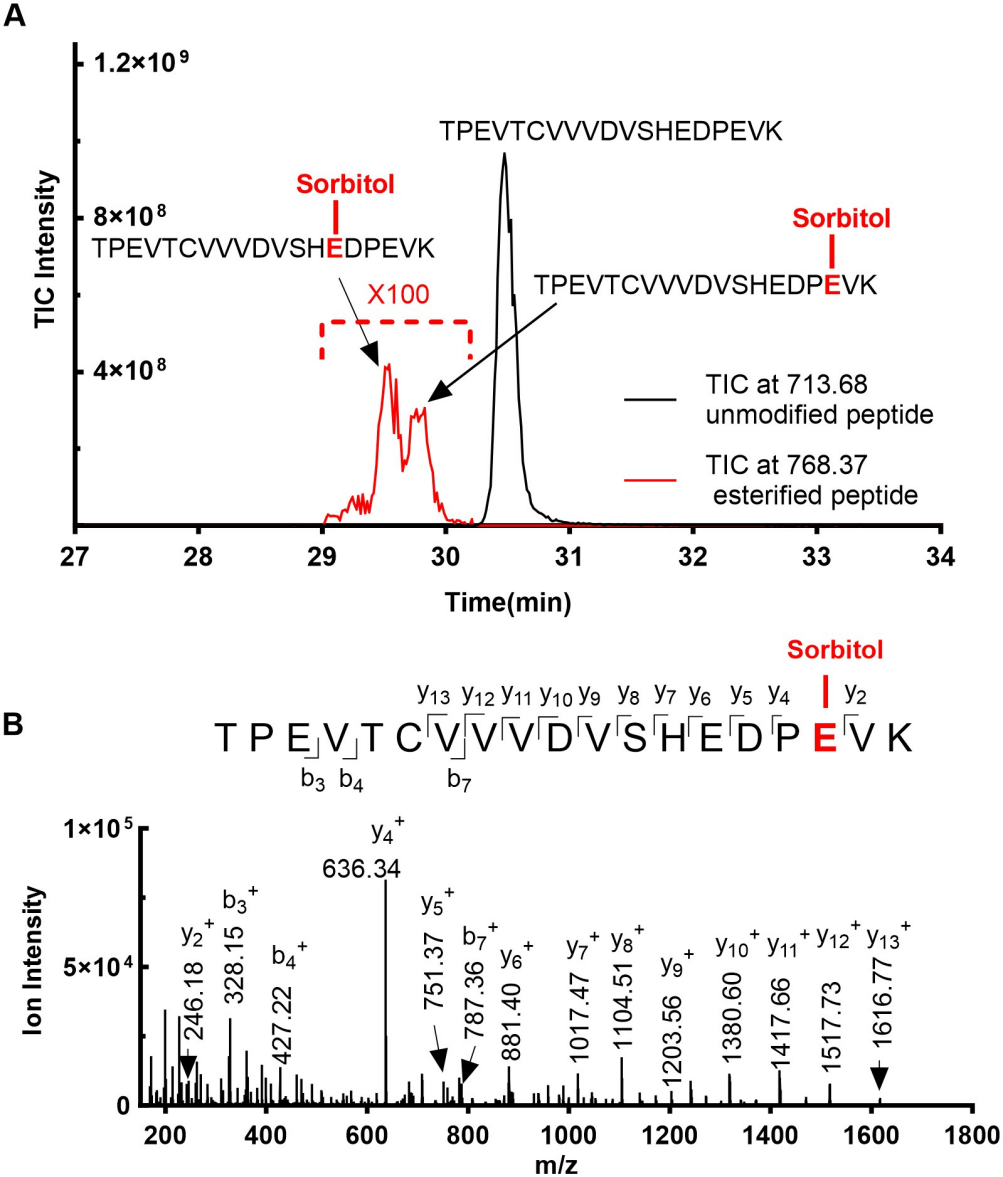
Total Ion Chromatogram (TIC) and MS/MS spectra showing the site- specific identification of esterified glutamic acid residues within the tryptic peptide HC 263–281. **(A)** Total Ion Chromatogram showing distinct esterification peaks with identical m/z eluting prior to the unmodified peptide peak. **(B)** MS/MS spectrum of E279 esterification within the tryptic peptide HC 263–281. Despite sharing an identical parent mass, this peptide possesses a distinct y_4_^+^ -y_5_^+^ ion series compared to the E276 esterified residue in HC 263–281 (Fig 2B).

In the case of the two peptides with three modified residues, the chromatographic resolution was sufficient to distinguish the esterified peptides from their respective unmodified forms. For HC 263–281 (TPEVTCVVVDVSHEDPEVK), the esterified peptide elutes prior to the unmodified peptide across 2 distinct peaks (Fig 3A). MS/MS analysis confirmed the site of esterification in these two peaks as E276 and E279 in respective order (Figs 2B and 3B). E265 esterification however appears to be below the detection limit.

**Fig 4:**
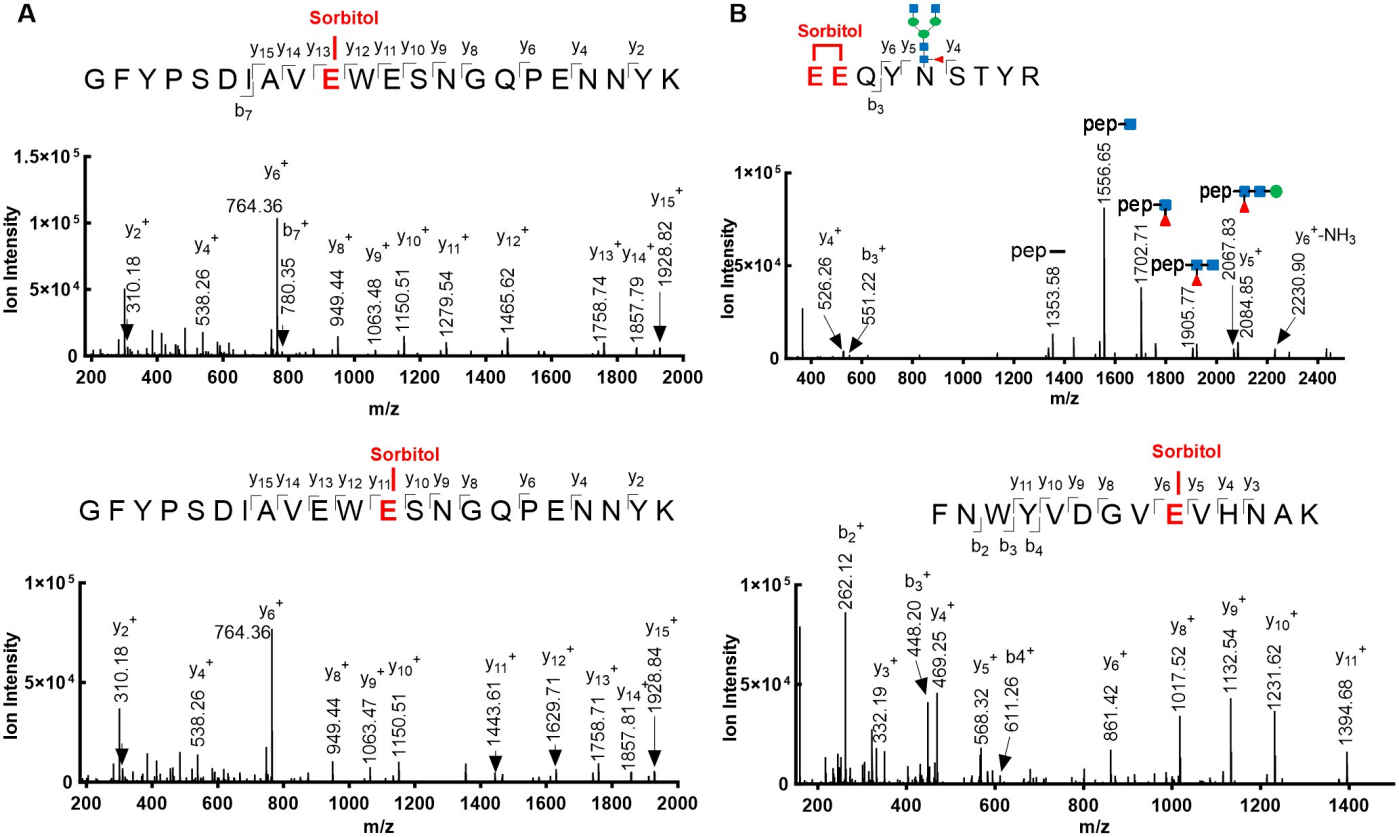
MS/MS spectrum of multiple esterified glutamic acid residues. **(A)** Two MS/MS spectra of HC 378–399 with identical m/z showing the esterification at E387 and E389 differentiated by distinct y_11_^+^ and y_12_^+^ ions. **(B)** MS/MS spectra of tryptic peptide HC 300–308 showing esterification at either E300 or E301. The highest intensity singly charged ion at m/z 1556.65 is a MS/MS fragment of a peptide containing the N-acetylglucosamine remnant of a liberated N- glycan. Color code: blue square-N-acetylglucosamine; red triangle-fucose; green circle-mannose. **(C)** MS/MS spectrum of HC 282–295 demonstrates E290 as the site of esterification.

The same phenomenon was observed in HC 378–399 (GFYPSDIAVEWESNGQPENNYK). In this case, two of the three glutamic acids were identified to be esterified by MS/MS in Fig 4A. Two peaks corresponding to the E389 and E387 esterification modifications eluted sequentially prior to the unmodified peptide. No evidence for E395 esterification modification was observed.

In each of the two peptides (HC 300–308 and HC 99–128) containing two glutamic acids, the residues are adjacent to each other. Due to their proximity, unambiguously assignment of which glutamic acid was esterified was not possible. Nonetheless, MS/MS spectra for HC 300–308 in Fig 4B reveals that esterification occurred within one of the glutamic acid residues. Three esterified tryptic peptides containing a single glutamic acid residue were identified, as demonstrated by the MS/MS spectrum in Fig 4C for the HC 282–295. Overall, eleven esterified glutamic acid residues from seven tryptic peptides were identified.

It was also observed that all seven peptides with esterified residues eluted earlier than their unmodified tryptic peptides. This phenomenon indicates a change in the overall hydrophobicity of the peptide. The large size of the sorbitol molecule within the glutamic acid side chain may induce a structural disruption, which opens the peptide to increase the solvent exposure and add more hydrophilic surfaces.

### Esterification levels correlate with incubation time and sorbitol concentration

The levels of glutamic acid esterification were calculated by measuring areas of an esterified peptide extracted ion chromatogram (XIC) peak relative to that of the corresponding un-esterified peptide. For cases in which an esterified peptide was also modified with iodoacetamide or N-glycosylation, comparative intensities were measured against the unmodified peptide with cysteine alkylation or a G0F N-glycan moiety respectively. The percentage of esterification for individual glutamic acid residues from the sorbitol 4-week (270 mM) and sorbitol (270 mM) + sorbitol ^13^C6 (270 mM) 4-week samples, as well as its location and amino acid sequence are summarized in Table 1. The increase in esterification levels all corresponded to a sample’s incubation time (from 0 to 4 weeks) as well as the total concentration of sorbitol. Levels of esterification were low or undetectable at prior to incubation while intensities ultimately increased several fold when incubated for 4-weeks. Also, esterification levels increased with total sorbitol concentration.

**Table 1:** Summary of observed esterified glutamic acid residues, location and percent esterification.

Tryptic peptides with esterification		Esterification percentage at 4-week sample	
Sequence	Site	Sorbitol sample ^a^	Sorbitol + Sorbitol ^13^C6 sample ^b^
TPEVTCVVVDVSH**E**DP**E**VK	HC E276	0.3%	0.9%
	HC E279	0.2%	0.8%
GFYPSDIAV**E**W**E**SNGQPENNYK	HC E387	0.1%	0.6%
	HC E389	0.2%	0.4%
**EE**QYNSTYR	HC E300/E301^c^	0.8%	2.8%
XXXXXXXX**EE**XXXXXXXXXXXXXXXXXXXX	HC E107/E108^c^	0.4%	1.0%
FNWYVDGV**E**VHNAK	HC E290	0.2%	0.5%
V**E**IK	LC E105	0.2%	0.7%
XX**E**XXXXXXXXXXXXXXX	LC E27	0.1%	0.4%

HC: Heavy Chain; LC: Light Chain. The location of esterified glutamic acid residues is shown and labeled in bold for each peptide. Esterification percentages were calculated from the integrated XIC peaks of the esterified peptides relative to their corresponding unmodified peptides.

^a^4-week sample with 270 mM sorbitol.

^b^4-week sample with 270 mM sorbitol and 270 mM ^13^C6 sorbitol.

^c^Insufficient MS/MS coverage to distinguish which glutamic acid residue was modified.

After 4 weeks of incubation, esterification levels for E276 and E279 from HC 263–281 reached 0.3% and 0.2% respectively. It should be noted that the sorbitol (^13^C6) 4-week sample contained twice the sorbitol as the 4-week stability sample, which is reflected in the observation of a several fold elevation in esterification for the E276 and E279 sites, reaching 0.9% and 0.8% respectively. A similar trend was observed for E387 and E389 (HC 378–399) as well as each of the three peptides containing a single glutamic acid (E290 from HC 282–295, E105 from LC 104–107 and E27 from LC 25–42). All five of these glutamic acid residues exhibited esterification levels of approximately 0.1%-0.2% in the 4-week stability sample and approximately 0.4–0.7% in the sorbitol (^13^C6) 4-week sample respectively.

For each of the peptides containing two esterified glutamic acids (HC 300–308 and HC 99–128) the percentage of esterification was calculated as the sum of each of the two esterified glutamic acid residues. Esterification levels for HC 99–128 and HC 300–308 were 0.4–0.8% in the 4-week stability sample and increased to 1.0–2.8% in the sorbitol (^13^C6) 4-week sample.

**Fig 5:**
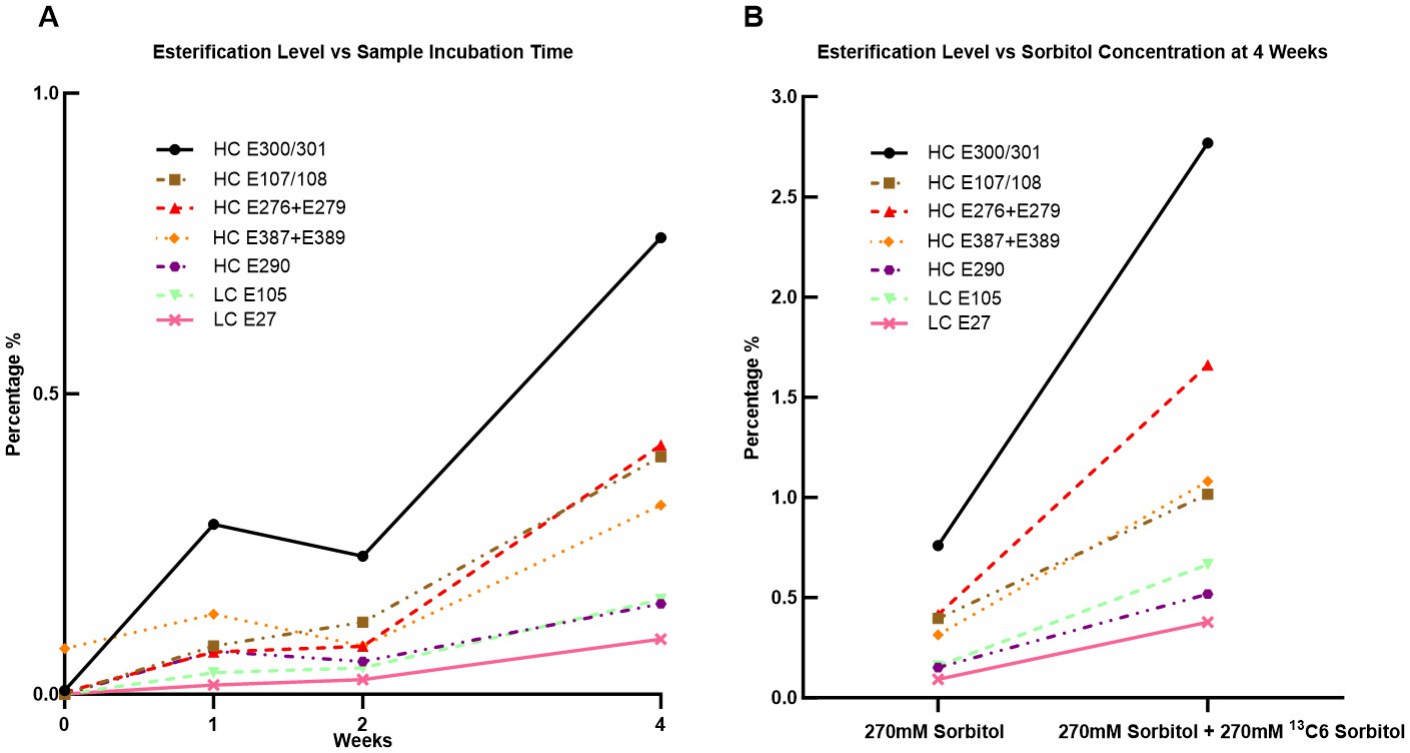
Esterification levels in each modified peptide. **(A)** The esterification percentages of all modified peptides from the 0 week, 1 week, 2 weeks and 4 weeks samples increased over time. **(B)** The percentage of esterification for all peptides in the 4-week sample with 270mM sorbitol and 270mM ^13^C6 sorbitol was two to six times higher than in the 4-week sample with 270mM sorbitol alone, representing a two-fold increase in sorbitol concentration.

For HC263-281 and HC 378–399, esterification levels were also obtained by adding the modification percentages for each glutamic acid for a given peptide. All seven peptides’ esterification percentages are shown in Fig 5. For each modified peptide, the esterification levels increased across the 4 weeks of incubation (Fig 5A). Commensurate with incubation times, an increase in esterification also appears to correlate with the sorbitol concentrations (Fig 5B). This effect was most notable for peptide HC 300–308, as the esterification percentage increased from 0% to 0.8% in the 4 week stability sample, while reaching 2.8% in the sorbitol (^13^C6) 4-week sample.

## Discussion

Glutamic acid esterification was observed for mAb X stressed stability samples incubated with sorbitol. Esterification was observed on both the heavy and light chains, spanning both conserved and unconserved regions. No aspartic acid residues were found to be esterified despite having a free carboxylic acid moiety. This may be due to steric hindrance from the aspartic acid side chain as it is one -CH_2_- group shorter and thus less flexible than the glutamic acid side chain. Sugar alcohol excipients, such as mannitol and glycerol are structurally similar and are reported to react with glutamic acid via their alcohol (-OH) groups by esterification [18, 19]. These excipients were not assessed in this study.

In the presence of sorbitol, the esterification of glutamic acid was found to correlate with incubation time and sorbitol concentration. Though the absolute level of esterification at an individual glutamic acid is relatively low, the sum of all esterified glutamic acid residues is much larger, presenting a potentially detrimental PTM that may impact the structure of the intact antibody. Total esterification levels of an intact antibody (by summing all esterification events together across each of the heterodimers), reaches 5% in the 4-week stability sample. In the sorbitol (^13^C6) 4-week sample, which contained twice the amount of sorbitol as the 4-week stability sample, the esterification levels escalate to 16%. Therapeutic drugs stored at 4°C may have levels of glutamic acid esterification below detection. However, for formulations containing sugar alcohol excipients, it may be advisable to evaluate the levels of glutamic acid esterification alongside other PTMs such as deamidation or oxidation.

Although current observations indicate low levels of esterification per site, it is possible that higher levels could induce detectable change in the conformation or secondary structure of mAb X. Furthermore, a reduction in hydrophobicity was observed in esterified peptides via C18 reverse phase HPLC, suggesting earlier elution compared to unmodified peptides, hinting at a potential trend at larger scales.

Esterification may be of interest and importance due to two reasons: firstly, the modification of glutamic acid by adding a sorbitol molecule may open and disrupt the local intact antibody structure; and secondly, esterification of glutamic acid by sorbitol converts the acidic free carboxylic acid side chain to a neutrally charge ester, changing the antibody’s surface charge and thus potentially affecting the antibody’s structure, solubility and stability. Therefore, esterification of glutamic acid residues could be considered as an important stability indicator.
